# Transcriptomic profiling of *Melilotus albus* near-isogenic lines contrasting for coumarin content

**DOI:** 10.1038/s41598-017-04111-y

**Published:** 2017-07-04

**Authors:** Kai Luo, Fan Wu, Daiyu Zhang, Rui Dong, Zhichao Fan, Rui Zhang, Zhuanzhuan Yan, Yanrong Wang, Jiyu Zhang

**Affiliations:** State Key Laboratory of Grassland Agro-ecosystems, College of Pastoral Agriculture Science and Technology, Lanzhou University, Lanzhou, 730020 China

## Abstract

Coumarin and its derivatives are widely used as fragrances in industrial products and have medical value. The goal of the present study was to discover genes and pathways related to coumarin biosynthesis in *Melilotus albus* using transcriptome analysis. The genes of five *M. albus* near-isogenic lines (NILs) that had different coumarin content and β-glucosidase activity according to the investigation of pedigree were quantified and then analysed by RNA-Seq. Using transcriptome analysis, differentially expressed genes (DEGs) were identified in two pairwise comparisons that differed in coumarin content as well as in two pairwise comparisons that differed in β-glucosidase activity. Gene expression pattern analysis suggested similar transcriptional trends in the genotypes with the same coumarin levels. Furthermore, the Kyoto Encyclopedia of Genes and Genomes (KEGG) database of DEGs was used to identify functional pathways associated with coumarin biosynthesis. We identified 111 unigenes, with several DEGs among them possibly being related to coumarin synthesis pathways. Unigenes encoding a hexokinase, an abscisic acid receptor, a phenylalanine ammonia-lyase (PAL) and two peroxidases particularly showed correspondence with the coumarin content of different genotypes. These results will contribute to a better understanding of the coumarin biosynthesis in *M. albus*.

## Introduction

Sweetclover (*Melilotus* spp.) is a forage crop belonging to the legume family and is native to Eurasia and North Africa^1, 2^. The genus is divided into approximately 19 species. Members of the *Melilotus* genus have adapted to extreme environments, such as drought and cold^2^, and can grow in moderately saline areas where traditional forage legumes cannot^3^. The nitrogen fixation rate of *Melilotus* species is superior to other legumes, making it beneficial for crop rotations^4^. Furthermore, *Melilotus* is used as soil stabilizer, ground cover and nectar source in some countries^5, 6^. Certain member of the *Melilotus* genus, such as *Melilotus albus* has been reported as species with good forage productivity^7^. In addition to being an important forage crop, due to their coumarin contents, there is increased interest in the industrial and medicinal value of *M. albus* ^8^.

Coumarin can be found in several plant species, such as *Melittis melissophyllum* L.^9^, *Dipteryx odorata* Willd.^10^, and *Mikania glomerata* Spreng.^11^. Nair *et al*. found coumarin contents ranging from 0.08% to 1.39% of dry matter in 15 *Melilotus* species^12^, and a preliminary evaluation of 19 *Melilotus* accessions also reported that coumarin content could vary from 0.16–1.02% of dry matter^13^. Coumarins (1,2-benzopyrones) derived from the phenylpropanoid pathway can be classified into four categories: simple coumarins, furano-coumarins, pyrano-coumarins and pyrone-substituted coumarins^14^. Like other phenylpropanoid family members, many coumarin compounds have been reported to be stress-induced, multidefensive secondary metabolites, and coumarins have been reported to be phytoalexins, allelochemicals and insect-feeding deterrents in plants. For instance, similar to salicylic acid, scopoletin is involved in plant responses to stressors^15^. Constituent umbelliferone was identified as a stress metabolite of *Chamomilla recutita* ^16^. A study on the interaction of the plant parasites with resistant and susceptible sunflower cultivars reported that resistant sunflower accumulated higher levels of coumarins in the roots and excreted greater amounts of coumarins than did susceptible varieties in response to infection^17^.

Recently, these components continue to receive attention for their diverse bioactivities, including antioxidant^15^, anti-inflammatory^18^, antibacterial^19^, termiticidal and rodenticidal activities^20^. Coumarin and its derivatives are widely used as fragrances in perfumes, cosmetics and soaps^21^. These compounds also have medical value due to their therapeutic properties, including inflammation modulation, edema reduction and possible anticancer activity^22^. Moreover, a large number of studies focusing on the therapeutic and pharmacologic properties of coumarins have supported their therapeutic roles in AIDS and cancer treatments^23–25^. Indeed, pyrano-coumarins and furano-coumarins have been applied as anti-HIV and anti-tumour therapies^24^. Despite the benefits of coumarin for plants and humans, high concentrations of coumarin are also a major limiting factor in the use of *Melilotus* species. Coumarin has been associated with dicoumarol production, which is an anticoagulant, and high concentrations in forage or conserved fodder are undesirable for grazing animals^12^. Therefore, the success of forage cultivar development based on *Melilotus* species will depend on decreasing coumarin content.

Investigations of coumarin biosynthesis were conducted during the 1960s and ’70s with the help of tracer-feeding experiments^26^. Stoker and Bellis elucidated the general scheme of the coumarin biosynthetic pathway in *M. albus*, which generates coumarin from L-phenylalanine via the intermediates *trans*-cinnamate, *trans*-2-coumarate, *trans*-2-coumarate-β-D-glucoside and *cis*-2-coumarate^27^. The enzymes involved in each step and the enzymatic reactions have been identified^28–32^. However, to date, there is a general lack of gene information regarding the enzymes involved in the coumarin biosynthesis pathway. Recently, several branch pathways and enzymes catalysing coumarin-formation reactions in other plant species have been identified with the help of modern synthesis and molecular techniques. For example, tyrosine ammonia-lyase (TAL) activity, which has been detected in *Glycine max* ^33^ and other species, is considered to be functionally similar to that of phenylalanine ammonia-lyase (PAL). Kai *et al*. identified in *Arabidopsis thaliana* a 2-oxoglutarate-dependent dioxygenase (2OGD) and a feruloyl-CoA 6′-hydroxylase (F6′H1) that exhibit *ortho*-hydroxylase activity towards the feruloyl coenzyme^34^. Genes homologous to F6′H from *Ipomoea batatas* and *Rue graveolens* have also been cloned and functionally analysed as *ortho*-hydroxylases of cinnamate CoA thioesters^35, 36^. These studies also reported the possibility of the synthesis of three coumarins (esculetin, umbelliferone and scopoletin). Because some pathways might be confined to a taxonomic group, studies of coumarin biosynthesis in different plants will help to elucidate these pathways in nature.

At present, several molecular techniques are employed in the discovery of important biological information in plants. Our previous studies applied molecular markers to assess phylogenetic relationships^37^ and genetic diversity^38, 39^ among *Melilotus* species. Genetic variation for key agronomy traits and coumarin content was estimated during a *Melilotus* breeding programs^40^. With the development of next-generation sequencing technology, RNA-Seq has been used as an efficient approach to understanding transcriptome profiles. Compared to microarrays, RNA-Seq does not require prior sequence knowledge, and it also provides superior precise measurement of transcripts. Indeed, a number of transcriptomic studies involving gene annotation, transcript profiling and gene discovery have recently been carried out^41–43^. In the present study, we used Illumina RNA-Seq technology to analyze the transcriptome of *M. albus* based on fifteen cDNA libraries from genotypes with different coumarin levels and β-glucosidase activities. To our knowledge, this is the first comprehensive transcriptomic study for the global discovery of differentially expressed functional genes and pathways related to coumarin content in *M. albus*. The results also provide an important new bioinformatic resource for further identification of genes and gene functions in non-model plant systems.

## Results and Discussion

### Comparison of coumarin content and β-glucosidase activity in *M.albus* genotypes

**Figure 1:**
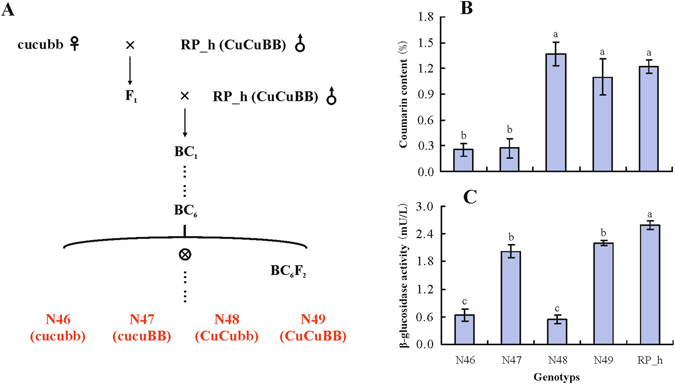
Details of *M. albus* genotypes used in deep sequencing. (**A**) Summary of the pedigrees of five genotypes, (**B**) and (**C**) The coumarin content and β-glucosidase activity in each genotypes, three individual replicates were performed for each genotype. The significant differences were analysis and alphabet indicated *P* value < 0.05. Cu/cu and B/b are two pairs of alleles affecting coumarin content and β-glucosidase activity, respectively.

To select the genotypes of *M. albus* for a comprehensive characterization of genes associated with coumarin biosynthesis, coumarin content and β-glucosidase activity were measured in five genotypes. As shown in Fig. 1, the coumarin contents in N48 and N49 were significantly (*P* < 0.05) higher than those in N46 and N47 as expected based on their genotypes. At the same coumarin level, N47 and N49 showed higher β-glucosidase activities than did N46 and N48, respectively, and RP_h had a coumarin content and β-glucosidase activity similar to that of N49. Goplen *et al*.^44^ studied the influence of two pairs of alleles, Cu/cu and B/b, upon the level and form of coumarin in *M. albus*. This study suggested that Cu/cu alleles determine high or low levels of coumarin and that the B/b alleles influence the form and type of coumarin^44^. A subsequent report demonstrated that the *o*-hydroxylation of cinnamic acid is a key step of coumarin biosynthesis that is influenced by Cu/cu alleles^45^. β-glucosidase activity is under the control of the B/b alleles and influences the transformation from bound coumarin to free coumarin^46^. In brief, plants of the CuCuBB genotype contain high levels of both free and bound coumarin. CuCubb leaves are also high in coumarin, but virtually all the substance is in the bound form; cucuBB and cucubb leaves both have low levels. Such comparison of contrasting coumarin contents at different β-glucosidase activity levels in *M. albus* might help to further understand coumarin biosynthesis.

### RNA-sequencing and *de novo* assembly of *M. albus* genotypes

RNA from five genotypes of *M. albus*, with three biological replicates, was used to construct cDNA libraries. A total of 490,655,520 raw reads were obtained, reflecting three biological replicates from five genotypes (N46, N47, N48, N49 and RP_h). After removing the adaptor sequences, low-quality sequences and the ambiguous nucleotides, a total of 453,181,574 clean reads remained, constituting over 67.99 GBase of data. The Q20 values were more than 94.60%, and the GC percentages of the five above *M. albus* samples ranged from 41.91% to 42.93%, respectively (Table 1). All of the clean reads were further pooled together and *de novo* assembled using Trinity^47^. Finally, a total of 154,458 transcripts of greater than 200 bp were obtained. The average length of the unigenes was 737 bp, and the N50 and N90 lengths were 1,297 and 281, respectively (Table S1). The size distributions of the unigenes and transcripts of *M. albus* are shown in Figure S1.

**Table 1:** Assessment of assembly quality for *M. albus* libraries of different genotypes.

Sample	Raw Reads	Clean reads	Clean bases	Error rate (%)	Q20 (%)	GC percentage (%)
N46_S1	36,176,224	33,640,042	5.05	0.02	95.12	42.36
N46_S2	29,235,058	27,116,128	4.07	0.02	94.80	42.62
N46_S3	33,407,970	30,839,890	4.63	0.02	94.81	42.51
N47_S1	34,375,458	31,815,210	4.77	0.02	94.95	42.51
N47_S2	29,447,642	27,242,772	4.09	0.02	94.78	42.68
N47_S3	29,704,900	27,297,328	4.09	0.02	94.60	42.75
N48_S1	30,321,602	27,994,636	4.20	0.02	95.06	42.74
N48_S2	32,703,906	30,244,572	4.54	0.02	94.93	42.53
N48_S3	34,530,430	31,962,230	4.79	0.02	95.02	42.61
N49_S1	29,580,194	27,368,482	4.11	0.02	95.11	42.64
N49_S2	33,381,510	30,688,264	4.60	0.02	94.97	42.93
N49_S3	31,450,096	29,068,470	4.36	0.02	94.80	42.78
RP_h_S1	41,333,864	38,058,638	5.71	0.02	95.18	41.91
RP_h_S2	31,107,038	28,474,496	4.27	0.02	94.68	42.55
RP_h_S3	33,899,628	31,370,416	4.71	0.02	95.11	42.59

Note: All the values were used RNA-seq data derived from three replicates in each genotype. The number of reads before and after quality trimming is given. Error rate: The sequence length multiplied by the number of sequencing. Q20: The percentage of bases with a Phred value > 20. GC percentage: The percentage of bases number of G and C.

### Gene functional annotation

Annotation of unigenes was performed by BLAST querying (E-value ≤ 10^−5^) against different databases (Figure S2, Table S2). A total of 42,878 unigenes were annotated, with significant BLAST results from the Nr (NCBI non-redundant protein sequences) database. Of these unigenes, 18,904 unigenes were shared by all near-isogenic lines (NILs) and their recurring male parent; these unigenes might be derived from the recurring parent in all genotypes (Table S3). Similarity distribution results showed that 67.3% of the matches were of high similarity, ranging from 80% to 100% similarity, as reported in the BlastX results (Figure S3A). Further analysis of the matching sequences revealed closest matches with *Medicago truncatula* for 65.6% of the sequences. The second-closest matches were with sequences from *Cicer arietinum*; 2.9%, 1.5% and 0.9% of the sequences showed closest matches with sequences from *G*. *max*, *Vitis vinifera* and *Phaseolus vulgaris*, respectively (Figure S3B). The E-value distribution results showed strong similarity for 48.0% of the homologous sequences (smaller than 1e–60) (Figure S3C).

To identify the functional categories of the annotated unigenes, GO (Gene Ontology), KOG (euKaryotic Ortholog Group), and KEGG (Kyoto Encyclopedia of Genes and Genomes) were used to classify the unigenes annotated by known proteins. In total, 33,537 *M. albus* unigenes were allocated to 46 functional groups belonging to three GO categories: “Cellular Component” (53,676), “Biological Process” (82,345) and “Molecular Function” (41,048), respectively (Figure S4A, Table S4). A total of 18,499 unigenes were annotated and grouped into 26 KOG categories and the cluster related to “general function prediction only” (3,282, 17.32%) was the largest group (Figure S4B, Table S5). Furthermore, 17,995 unigenes were assigned to five main categories, which include 32 sub-categories and 277 KEGG pathways (Figure S5, Table S6). For metabolism sub-categories, the biosynthesis of secondary metabolites presented the most predominant pathways; these were classified into 14 subcategories, including phenylpropanoid biosynthesis, flavonoid biosynthesis, and tropane, piperidine and pyridine alkaloid biosynthesis (Figure S6). The genes involved in these pathways may provide a resource for researching specific biochemical and development processes in *M. albus*.

### Differentially expressed genes (DEGs) analysis

**Figure 2:**
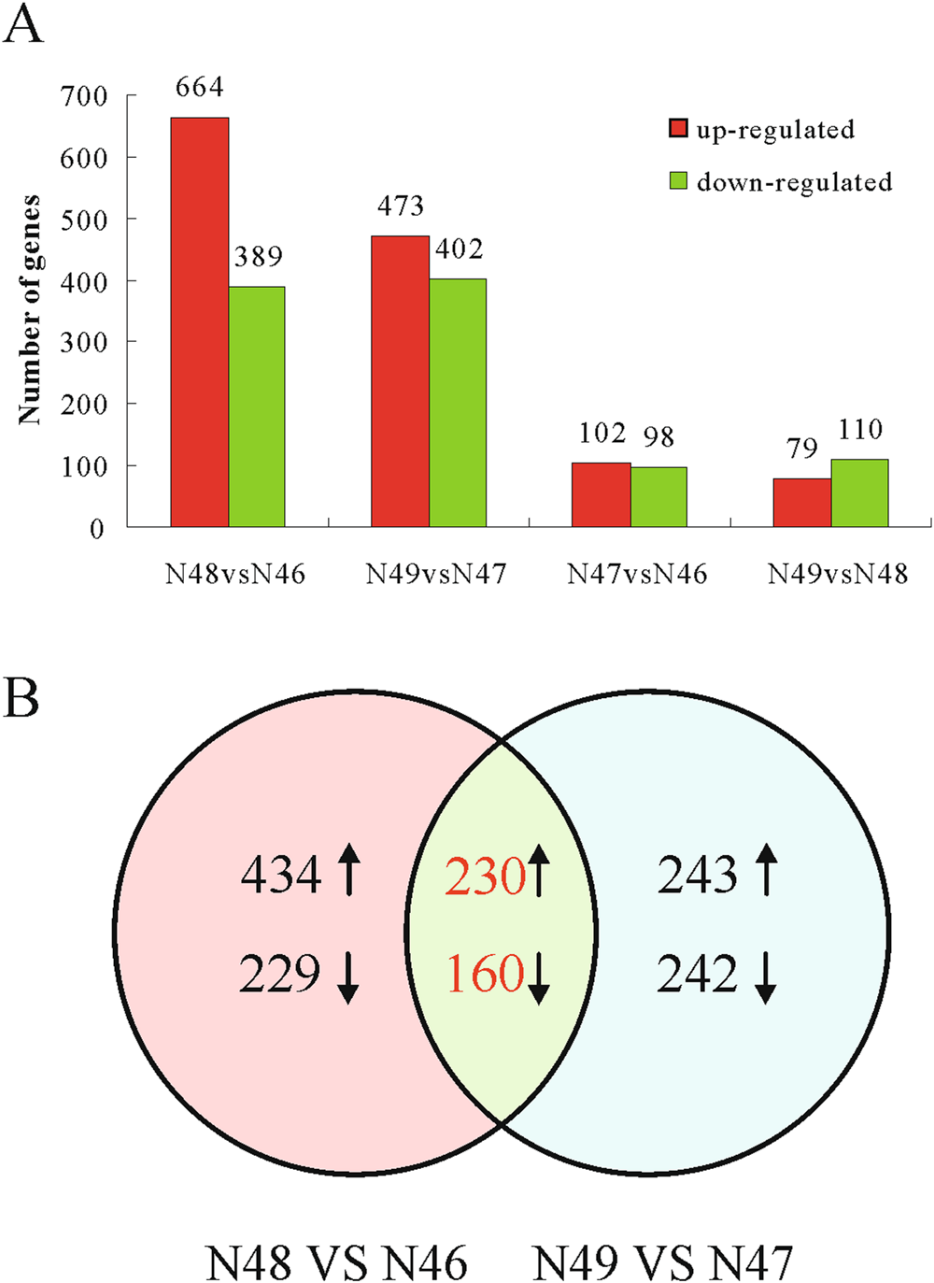
Differential gene expressions of *M. albus*. (**A**) The number of up- and down-regulated genes in comparisons of N48 vs N46; N49 vs N47; N47 vs N46 and N49 vs N48. (**B**) The Venn diagrams of DEGs from N48 vs N46 and N49 vs N47. The numbers marked in the diagram are the number of common genes between the two sets (log_2_ fold change ≥ 1 and padj ≤ 0.05).

We required a padj of 0.05 or less to identify DEGs by using DESeq. Comparisons of gene expression between N48 and N46 (different coumarin expression at a low β-glucosidase activity level) showed 1,053 DEGs. The number of the DEGs is greater than that in the high-β-glucosidase-activity genotypes (N49 vs N47), which showed 875 DEGs. In total, 200 and 189 unigenes were significantly differentially expressed between N47 vs N46 and between N49 vs N48, respectively. The numbers of up-regulated and down-regulated unigenes are shown in Fig. 2A. When comparing among the four pairwise comparisons, we discovered that there were many more differentially expressed genes in the comparisons of coumarin level (N48 vs N46 and N49 vs N47) than in the comparisons of β-glucosidase activity (N47 vs N46 and N49 vs N48), suggesting that regulation of coumarin biosynthesis is complex. We also found 230 up-regulated and 160 down-regulated unigenes that overlap between N48 vs N46 and N49 vs N47. These genes are likely involved in coumarin biosynthesis (Fig. 2B, Table S7).

**Figure 3:**
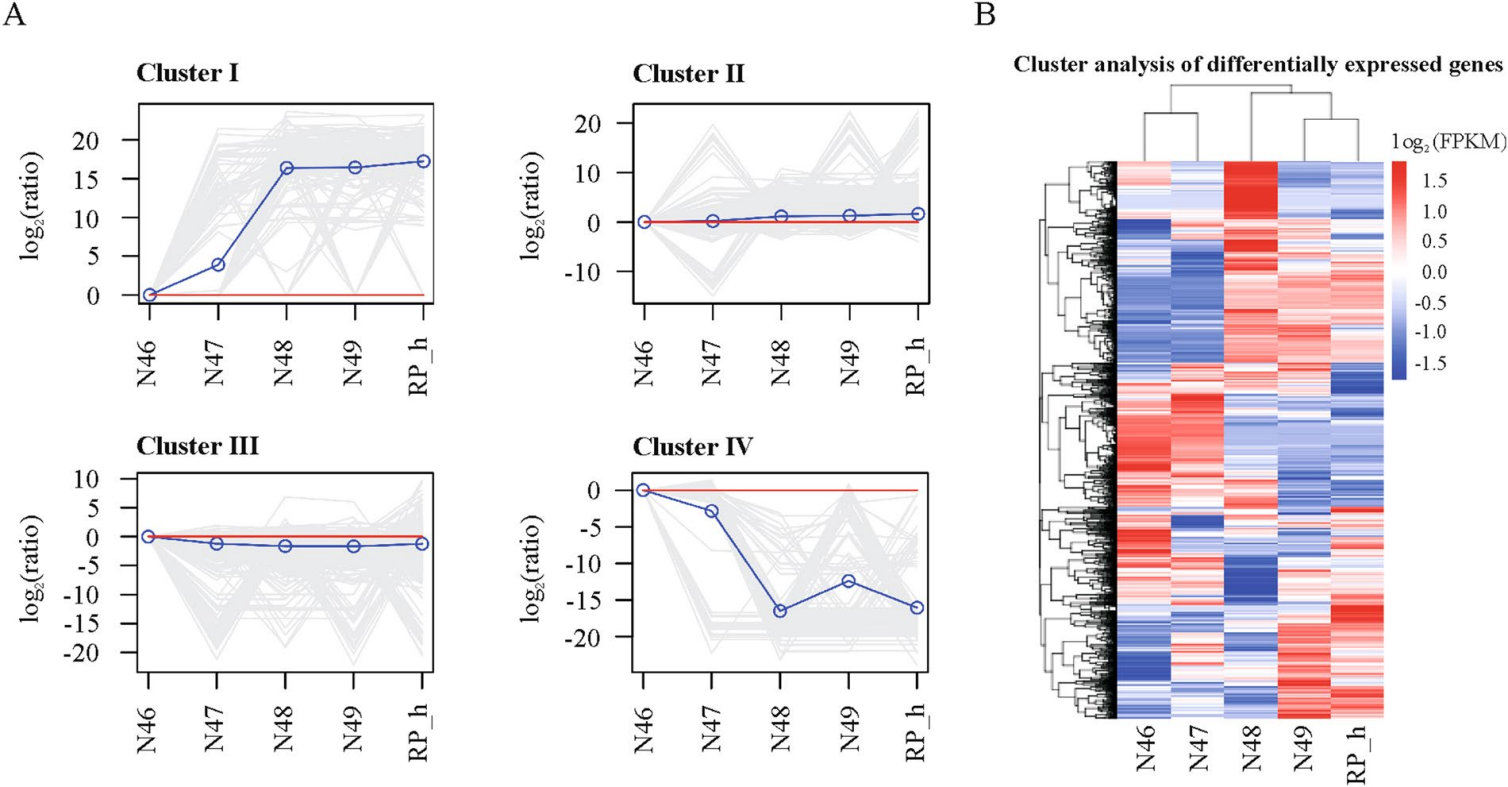
Cluster analysis of DEGs among five *M. albus* genotypes. (**A**) K-means clusters of the gene expression. All differentially expressed unigenes were divided into 4 distinct expression profiles. The x-axis represents the different genotypes. The y-axis gives the degree of fold change observed for the each given genotype vs the N46 (‘reference’), presented as the log_2_ (ratio) value. The blue lines show model expression profiles. The gray lines represent individual gene expression profiles. The red lines show the log_2_ (ratio) value of N46 (‘reference’). (**B**) Heat-map showing the expression of all DEGs, using RNA-seq data derived from mean value of three replicates in each genotype based on log_2_ (FPKM) values. Intensity of color indicates expression levels. Similarity between genotypes and unigenes with hierarchical clustering is shown above and the left of the heatmap, respectively.

To assess genotype-specific trends in gene expression, we performed a K-means cluster analysis using normalized expression values from each individual replicate of five different genotypes with contrasting coumarin contents. A total of 2,095 DEGs and four distinct clusters with similar expression patterns were produced (Fig. 3A). The gene number in each pattern ranged from 115 to 959; the lists of all DEGs in the four clusters are presented in Table S8. Cluster I contained genes preferentially expressed in high-coumarin genotypes; cluster IV comprised transiently up-regulated genes in low-coumarin genotypes. In contrast, cluster II and cluster III showed no significantly different gene patterns among the five genotypes. Interestingly, we found that the *M. albus* genotypes with the same coumarin levels represented similar gene expression patterns, indicating that coumarin levels should be dynamic due to changing expression via a series of gene regulation. This is also supported by the gene expression pattern shown in the heatmap (Fig. 3B), which shows that the genotypes clustered together according to their coumarin levels.

As shown in Fig. 3A, cluster I and cluster IV were of particular interest, as they identified unigenes that might be related to coumarin biosynthesis; a substantial number of unigenes in the two clusters matched with the hypothetical and/or uncharacterized proteins (Table S8). The putative functions of these genes, some of which are factors in both secondary metabolite biosynthesis and signal translation, transcription factors and members of the P450 family, might provide information to better understand their relationship with coumarin biosynthesis. Although the function of these genes appears clear, the main difficulty in such analyses is not in the identification of putative related DEGs but rather in the interpretation of how the genes may interact^48^. Clusters I and IV also presented a number of DEGs of unknown and/or unclassified function, and it is possible that these up- and down-regulated genes indirectly contribute to coumarin biosynthesis or are indirectly involved in coumarin-related regulatory networks. However, confirming this hypothesis would require a detailed exploration of their role in plants. To date, several studies have applied K-means clustering for analysing transcriptomes^48, 49^.

### Functional analysis of DEGs

**Figure 4:**
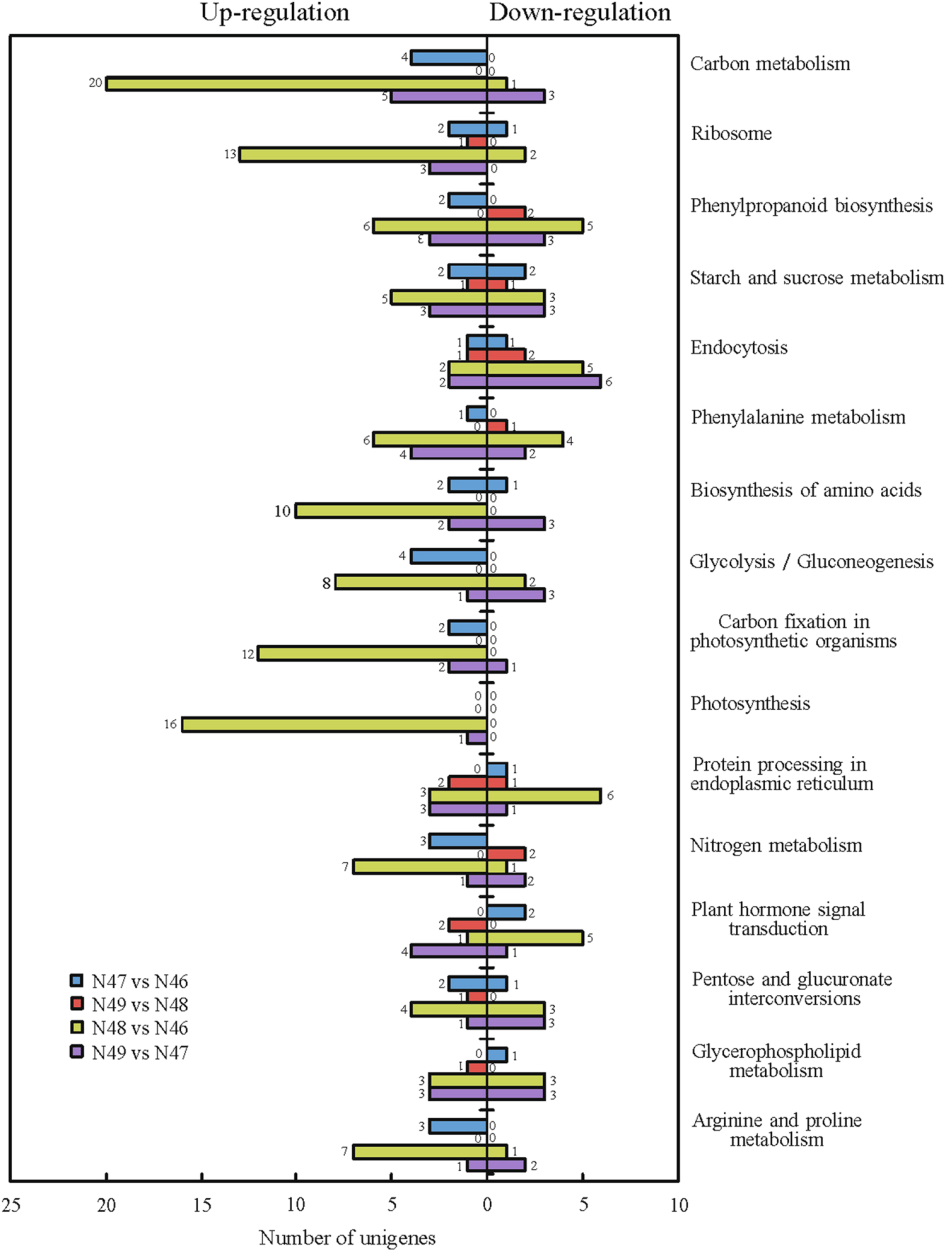
Functional categorization of genes differentially expressed in four comparisons of N48 vs N46; N49 vs N47; N47 vs N46 and N49 vs N48 based on Kyoto Encyclopedia of Genes and Genomes (KEGG) classification. Down-﻿ ﻿regulated (right) and up-regulated (left) unigenes were quantified. The numbers on the top of bar chart indicate the number of unigenes that were enriched in the KEGG pathway with Q value ≤ 0.05.

The KEGG database was used to further understand the biological functions and pathways of DEGs. The numbers of up-regulated and down-regulated unigenes of four pairwise comparisons assigned to KEGG categories are shown in Fig. 4.

**Figure 5:**
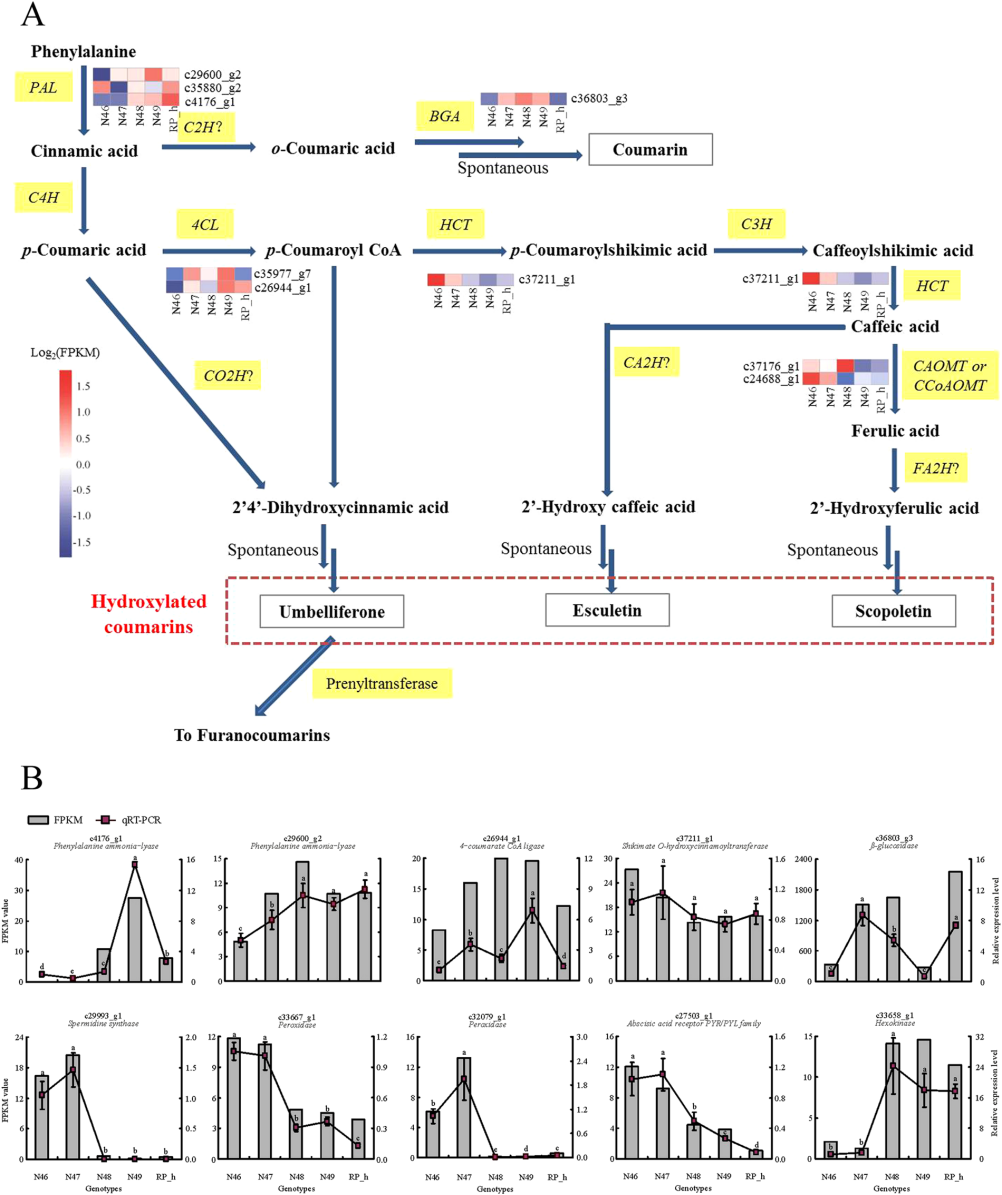
Expression patterns of genes involved in coumarin biosynthesis. (**A**) Simplified diagram depicting the biosynthesis pathway of coumarins in plant adapted from Bourgaud *et al*.^74^. Yellow box indicate that the enzyme involved in this pathway. Enzymes assigned by a question mark are hypothetical. Intensity of color indicates expression levels. Abbreviations: PAL, phenylalanine ammonia-lyase; C2H, cinnamic acid 2-hydroxylase; BGA, β-glucosidase; C4H, trans-cinnamate 4-monooxygenase; 4CL, 4-coumarate–CoA ligase; HCT, shikimate *O*-hydroxycinnamoyltransferase; C3H, coumaroylquinate (coumaroylshikimate) 3′-monooxygenase; CA*O*MT, caffeic acid 3-*O*-methyltransferase; CCoA*O*MT, caffeoyl CoA *O*-methyltransferase; CA2H, caffeic acid 2-hydroxylase; CO2H, 4-coumaric acid 2-hydroxylase, FA2H, ferulic acid 2-hydroxylase. Dotted border shows three major hydroxylated coumarins. (**B**) Expression patterns of genes probably related to coumarin biosynthesis by RNA-Seq were validated by qRT-PCR. Gray histograms indicate transcript abundance change based on FPKM values according to RNA-Seq (left y-axis). Lines with standard error bar represents relative expression level determined by qRT-PCR from three biological replicates of each genotype using 2^−∆∆CT^ method (right y-axis). Values with the same letter are not significantly different (Duncan’s test, *P* < 0.05).

Two pairwise comparisons of N48 vs N46 (CuCubb vs cucubb) and N49 vs N47 (CuCuBB vs cucuBB), which have contrasting coumarin contents at different β-glucosidase activity levels, were used to investigate the predicted biological function that may be involved in coumarin biosynthesis. In our enrichment analysis, most of the DEGs encoding enzymes were involved in carbon metabolism and carbon metabolism-related pathways (including glycolysis/gluconeogenesis) in both N48 vs N46 and N49 vs N47. Higher percentages of these genes were found to be up-regulated in N48 vs N46 and in N49 vs N47. For example, c33658_g1, which encodes hexokinase, was up-regulated in high-coumarin genotypes (Fig. 5B). The activity of hexokinase is expected to be critical to the cellular levels of glucose and fructose, and the reactions catalysed by hexokinase lead to hexoses entering the glycolytic pathway. It has been shown that hexokinase decreases at the transcript level in *Lolium multiflorum* lines resistant to drought stress^50^. Hormones are molecules produced by plant cells in response to environmental stresses. Interestingly, there are several DEGs encoding enzymes classified as involved in plant hormone signal transduction. One gene (c27503_g1) encoding an abscisic acid receptor in the PYR/PYL family showed a particular correlation with coumarin content across the different genotypes (Fig. 5). The abscisic acid receptor PYR/PYL has shown potential to enhance plant drought resistance^51^. These results indicated that the process of coumarin biosynthesis may indirectly affect plant adaptation to biotic and abiotic stress by carbon metabolism and signal transduction. A KEGG pathway with a large number of significantly enriched DEGs involved with “Ribosome” suggests that coumarin biosynthesis may indirectly affect the rates of protein synthesis and ribosome production during the plant regrowth^52^. Coumarin derivation from the phenylpropanoid pathway is well known. As expected, “Phenylpropanoid biosynthesis” and “Phenylpropanoid metabolism” were highly enriched among the DEGs, further confirming the efficiency of the gene expression data by comparing genotypes contrasting for coumarin content.

Comparisons of N47 vs N46 (cucuBB vs cucubb) and N49 vs N48 (CuCuBB vs CuCubb) were used to identify KEGG functional groups related to β-glucosidase activity. The first three classifications in both N47 vs N46 and N49 vs N48 comparisons were “Starch and sucrose metabolism”, “Endocytosis” and “Nitrogen metabolism”, suggesting that they may contribute to the modulation of β-glucosidase activity. The numbers of annotated DEGs in N48 vs N46 and in N49 vs N47 were greater than those in N47 vs N46 and in N49 vs N48, respectively, suggesting that the regulation of coumarin biosynthesis is complex and that a large number of genes may be related to this biosynthesis pathway.

### Expression analysis of phenylpropanoid pathway genes

**Figure 6:**
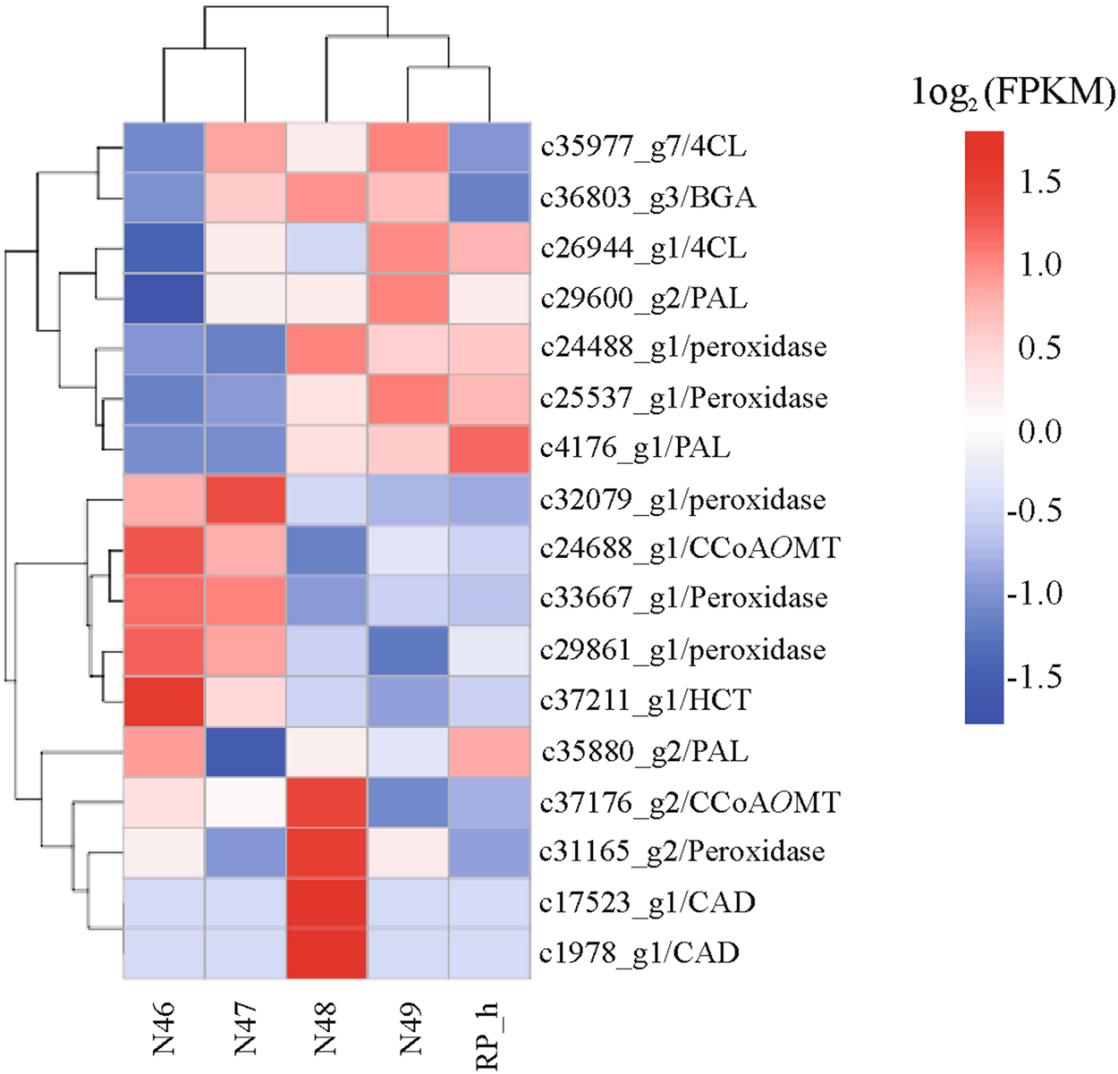
Heat-map showing expression patterns of phenylpropanoid biosynthesis genes differentially regulated among five *M. albus* genotypes. The gene expression was used RNA-seq data derived from mean value of three replicates in each genotype based on log_2_ (FPKM) values. Intensity of color indicates expression levels. Similarity between genotypes and unigenes with hierarchical clustering is shown above and the left of the heatmap, respectively. Abbreviations: 4CL, 4-coumarate–CoA ligase; BGA, β-glucosidase; PAL, phenylalanine ammonia-lyase; HCT, shikimate O-hydroxycinnamoyltransferase; CCoA*O*MT, caffeoyl CoA *O*-methyltransferase; CAD, coniferyl-aldehyde dehydrogenase.

Phenylpropanoids comprise a large group of natural products synthesized by enzymes of the phenylpropanoid pathway^53^. In our transcriptome data, 213 unigenes were assigned to the phenylpropanoid biosynthesis pathway based on KEGG pathway classification, with 17 DEGs in this pathway (Figure S6, Fig. 6, Table S9). Fig. 6 shows the regulation pattern of unigenes matching enzymes in the phenylpropanoid pathway. More than one unigene matched the same enzyme, indicating that these unigenes represent different members of a gene family or different fragments of a single transcript^54^. For example, PAL is the first enzyme in the phenylpropanoid pathway^55^, and 10 unigenes (including c14067_g1, c17925_g1 and c29600_g2) from the transcriptome were annotated as PAL. The highest number of unigenes (64 in total) that mapped to this pathway encode peroxidase. Class III plant peroxidase catalyses the plant-specific oxidoreduction between hydrogen peroxide (H_2_O_2_) and various reductants^56^ and is also associated with lignification, a normal process in plant growth that also occurs in response to environmental stress^57^. Six peroxidase unigenes were found to be differentially expressed among the five *M. albus* genotypes. Two peroxidase unigenes (c24488_g1 and c25537_g1) were up-regulated in high-coumarin genotypes compared with low-coumarin genotypes, and three genes (c32079_g1, c33667_g1 and c29861_g1) were down-regulated, suggesting that these genes might be involved in different physiological processes.

### The expression pattern of genes involved in coumarin biosynthesis

Among the four categories of coumarins, simple coumarins, furanocoumarins and pyranocoumarins are derived from the general phenylpropanoid pathway. The regulation of coumarin biosynthesis is complex. Major details remain unresolved, and many of the P450-dependent enzymatic steps are largely unknown. A simplified version of coumarin biosynthesis in plants that has been modified based on that of Bourgaud *et al*.^58^ is shown in Fig. 5. Simple coumarins consist of the simplest coumarin (shown as “coumarin” in this pathway), and its prevalent hydroxylated derivatives, umbelliferone, scoparone and esculetin are also presented in this pathway. The enzyme PAL has been identified to convert phenylalanine to cinnamic acid is the first enzyme in the coumarin biosynthesis pathway^27, 55^. The pathway from cinnamic acid to coumarin was characterized during the 1960s and ’70s. *o*-Hydroxylation is a key step in the coumarin biosynthesis pathway that converts cinnamic acid to *o*-coumaric acid. Unfortunately, the *o*-hydroxylation of cinnamic (or 4-coumaric) acid, being of pivotal importance for coumarins, remains to be a missing link in the phenylpropanoid biosynthesis network. In this pathway, endogenous β-glucosidase has been assigned to rapidly hydrolyse coumarinyl glucoside to yield coumarinic acid^46^.

In the pathway from cinnamic acid to other hydroxylated coumarins, *trans*-cinnamate 4-monooxygenase (C4H) adds a hydroxyl group to produce 4-coumarate acid, and CoA is linked by 4-coumarate-CoA ligase (4CL)^59^. 4-Coumaric acid is *o*-hydroxylated to 2,4-dihydroxycinnamic acid by a putative enzyme that is assigned to chloroplasts^60^. 4CL represent the key precursor in the main plant phenylpropanoid pathway, including the coumarin biosynthesis pathway. In addition, by converting 4-coumarate into 4-coumaroyl-CoA, 4CL is required for completion of coumarin synthesis^61, 62^. Shikimate *O*-hydroxycinnamoyltransferase (HCT) belongs to the large family of BAHD-like acyltransferases^63^ and is a key enzyme in the phenylpropanoid and lignin biosynthesis pathways. A study in Arabidopsis demonstrated that HCT gene silencing leads to significant changes in lignin content^64^. The role of HCT in coumarin biosynthesis has also been reported^61^. The coumaroylquinate (coumaroylshikimate) 3′-monooxygenase (C3H), which catalyses the 3′-hydroxylation of *p*-coumarate, revealed the origin from ferulic acid in Arabidopsis^34^. Caffeoyl CoA *O*-methyltransferase (CCoA*O*MT) participates not only in lignin biosynthesis but also in the biosynthesis of several soluble phenylpropanoids, including scopoletin^34, 65^.

Select unigenes involved in coumarin biosynthesis were further examined. A total of 111 unigenes encoding 8 enzymes were assigned to the coumarin pathway based on their KEGG pathway classification (Table 2), and RNA-Seq showed that some of these genes were differentially expressed among the five *M. albus* genotypes. These genes include three PAL genes, two 4CL genes, one β-glucosidase (BGA) gene, one HCT gene and two CCoA*O*MT genes (Figs 5 and 6).

**Table 2:** Summary of genes involved in coumarin biosynthesis in *M. albus*.

Gene	KO entry	EC no.	Enzyme	Number of unigenes
PAL	K10775	4.3.1.24	phenylalanine ammonia-lyase	10
BGA	K01188	3.2.1.21	β-glucosidase	58
4CL	K01904	6.2.1.12	4-coumarate–CoA ligase	19
HCT	K13065	2.3.1.133	shikimate *O*-hydroxycinnamoyltransferase	11
CCoA*O*MT	K00588	2.1.1.104	caffeoyl-CoA *O*-methyltransferase	8
C4H	K00487	1.14.13.11	trans-cinnamate 4-monooxygenase	1
C3H	K09754	1.14.13.36	coumaroylquinate(coumaroylshikimate) 3′-monooxygenase	1
C*O*MT	K13066	2.1.1.68	caffeic acid 3-*O*-methyltransferase	3

As one of the most studied enzymes, several PAL genes have been identified and characterized in many plant species, such as Arabidopsis^66^, tobacco^67^ and potato^68^. In our study, one PAL gene (c4176_g1) was up-regulated in N48, N49 and RP_h compared to that in N46 and N47 and showed a correlation with coumarin content, indicating its potentially important role in coumarin biosynthesis (Figs 5 and 6). In addition, high expression of another PAL gene (c29600_g2) was detected in the high-coumarin genotypes with similar β-glucosidase activities. Similarly, high expression of one 4CL gene (c26944_g1) was detected in the high-coumarin genotypes with similar levels of β-glucosidase activity. These two genes (c29600_g2 and c26944_g1) may play a role in coumarin biosynthesis under similar β-glucosidase activities. Increasing the activity of PAL to a level greater than that normally found in several species leads to a corresponding increase in coumarin content^69^. This could be caused by *cis*-cinnamic acid synthesis from overproduced *trans*-cinnamic acid, which is needed for synthesis of coumarin. Higher expression of the 4CL gene could shift more activity towards *p*-coumaric acid, resulting in the accumulation of umbelliferone and scopoletin. Three 4CL genes from the traditional Chinese medicine plant *Peucedanum praeruptorum* had higher transcript abundances in the roots than in the stems and leaves, which is in agreement with higher coumarin content in roots^70^. We also found differential expression of one PAL and one 4CL gene among the genotypes, but there was no corresponding change in coumarin level. This is most likely because different homologous genes encoding the same enzyme may differ in function. It has been demonstrated that C4H catalyses the key step of cinnamic to *o*-coumaric acid, and is necessary for umbelliferone formation^71, 72^. Only one unigene corresponding to C4H showed no differential expression among the genotypes, indicating that C4H is not the main reason for the observed changes in coumarin, despite the initial step of C4H in the coumarin pathway. In addition to coumarin biosynthesis, C4H constitutes the P450 enzyme most studied in several branch pathways, such as the flavonoid and lignin biosynthesis pathways^73, 74^. The expression pattern of the DEGs HCT and CCoA*O*MT were similar, with higher expression in the low-coumarin genotypes than in the high-coumarin genotypes. These results indicate that down-regulation of these two genes might reduce the ratio of scopoletin to total coumarin or lignin content in high-coumarin genotypes.

PPI (Protein-Protein Interaction) network visualization analysis was used to identify potential regulators of the DEGs and to predict regulatory interactions/relationships. The DEGs in N48 vs N46 in a pairwise comparison contrasting for coumarin biosynthesis were selected for coexpression and PPI network visualization analysis (Figure S7; Table S10). Furthermore, the computed coexpression relationships between genotypes that differed in coumarin content identified the coumarin biosynthesis related genes PAL, 4CL, CCoA*O*MT and HCT. These genes and their computationally predicted interactions were arranged together and are shown in yellow color; these genes include two PAL genes (c4176_g1 and c29600_g2), two 4CL genes (c26944_g1 and c35977_g7), one CCoA*O*MT gene (c37176_g2), one HCT gene (c37211_g1), four peroxidase genes (c32079_g1, c25537_g1, c33667_g1 and c29861_g1), one naphthoate synthase gene (c36645_g3) and one chalcone synthase gene (c6231_g1). This result indicated that there is a complex relationship within genes related to coumarin. The potentially important role of peroxidase in coumarin biosynthesis is also shown. The relationships between naphthoate synthase, chalcone synthase and coumarin biosynthesis-related genes need to be verified in future studies.

### Validation of RNA-Seq data by qRT-PCR

To validate the assembly and annotation of the RNA-Seq data, quantitative real-time PCR (qRT-PCR) was performed on thirty DEGs randomly selected from the expression profile data and ten unigenes likely associated with coumarin biosynthesis (Table S11; Fig. 5). The qRT-PCR expression patterns and FPKM values of these unigenes are shown in Table S11. Overall, the qRT-PCR values were highly correlated with the RNA-Seq results, confirming the reliability of the transcriptome and expression profile data.

## Conclusions

*M. albus* is one of the most important legume plants. Although it is widely used owing to its coumarin production, there is limited genomic information to date. This is the first report of the application of transcriptomic data to elucidate the regulation of coumarin biosynthesis in *M. albus*. In summary, we sequenced the transcriptome of *M. albus* and investigated genes associated with coumarin biosynthesis and the relationship with coumarin content. Our transcriptome analysis revealed that 438 unigenes classified into 14 pathways are involved in the biosynthesis of secondary metabolites. Based on KEGG classification of DEGs, functional pathways associated with coumarin biosynthesis were further identified. We found 111 unigenes associated with coumarin biosynthesis pathways, including several DEGs such as three PAL genes, two 4CL genes, one BGA gene, one HCT gene and two CCoA*O*MT genes. Among the important functional groups, unigenes encoding a hexokinase, an abscisic acid receptor, a PAL and two peroxidases particularly showed correspondence with the coumarin content of different genotypes, suggesting their potential key role in coumarin biosynthesis. This work provides valuable resources for bioengineering and *in vitro* synthesis of coumarin for potential industrial product development and medical research.

## Materials and Methods

### Plant materials

Cu/cu and B/b are two pairs of alleles affecting coumarin content (more accurately, o-hydroxycinnamic acid beta-D-glucoside) and β-glucosidase activity, respectively. Fig. 1 is a summary of the pedigrees of all near-isogenic lines (NILs) derived from an initial cross of cucubb biennial plants × CuCuBB plants of PI 165554. The cucubb segregates were then successively backcrossed six times to the PI 165554 to obtain genotypes N46 through N49, which differ in coumarin content and β-glucosidase activity^75^. The plants of cucubb biennial *M. albus* were representative of several F6 lines that have been derived from a single, doubly hererozygous plant. The original cross from which this F6 plant was derived involved a cucuBB plant as the female parent and a CuCubb plant as the male parent^76^. Line N46 through N49 is a set of four lines represented all possible homozygous combinations of the Cu/cu and B/b alleles (Fig. 1).

Four NILs of *M. albus* (N46, N47, N48 and N49) and the recurrent male parent (PI 165554), which used in this experiment, were selected from the National Plant Germplasm System (NPGS). In this study, PI 165554 is referred to as RP_h. Five genotypes were planted in a greenhouse in 25 cm plots containing agricultural soil, with a photoperiod of 16 h light, 26 °C/8 h dark, 18 °C. The agricultural soil (0–30 cm) from Yuzhong used in this experiment is loessal soil, with pH 7.1, total N of 0.746 g/kg and total P of 0.759 g/kg, respectively. The plants were irrigated with water once a week. In the flowering stage, the 3rd–4th compound leaves from the top were harvested for RNA isolation. Three *M. albus* plants from each genotype as independent biological replicates were wrapped in tinfoil and immediately frozen in liquid nitrogen. All leaves were stored at −81 °C until further processing. Further, the remaining fresh leaves of the same samples were harvested for determination of coumarin content and β-glucosidase activity.

### Assay of coumarin content and β-glucosidase activity

For the coumarin assay, samples from each replicate of five genotypes were ground in a mill to pass through a 1 mm screen. Microwaves assisted extraction, with the main advantages of both the considerable reduction in time and the smaller solvent consumption, has been demonstrated as an efficient method for coumarin extraction by Martino *et al*.^77^. In this study, microwave assisted extraction was performed on a Microwave apparatus (Hechuang, Jiangsu, China) and setting the microwave at 300 W power. The powdered material (0.1 g) was placed into an extraction vessel and microwave extracted twice with 1 ml of 60% ethanol at 30 °C for 30 min. Coumarin was quantified by high-performance liquid chromatography (HPLC) using an Agilent 1100 series system (Agilent, Santa Clara, USA) equipped with a diode array detector (DAD) detector (λ = 310 nm) and an Agilent-XDB C18 column (4.6 × 150 mm,5 μm particle size). The mobile phase consisted of 65:35 methanol:water. The flow rate was set at 1.0 mL·min^−1^, and an injection volume of 20 µL at 35 °C was used^78^. Before injection, each sample was filtered through a 0.45µm GHP membrane to remove solid residue. For β-glucosidase activity measurement, an enzyme-linked immunoassay assay (ELISA) was performed with a β-glucosidase activity assay kit (Meilian Bio Co., Ltd, Shanghai, China) following the manufacturer’s instructions.

### RNA isolation and library preparation for transcriptome analysis

Total RNA of six leaves from three samples in each genotype was isolated as three biological replications using an RNAprep pure Plant RNA Purification Kit (Tiangen Biotech, Beijing, China). The quality and quantity of total RNA were analyzed using an UltrasecTM 2100 pro UV/Visible Spectrophotometer (Amersham Biosciences, Uppsala, Sweden) and by gel electrophoresis. For each genotype, high-quality RNA from three replications was used for cDNA library construction and Illumina deep sequencing.

A total amount of 3 µg RNA per sample was used as input material for the RNA sample preparations. Fifteen cDNA libraries were generated using NEBNext^®^ Ultra™ RNA Library Prep Kit for Illumina^®^ (NEB, USA). RNA detection, cDNA library construction and Illumina deep sequencing were performed following the methods by the Beijing Novogene Biological Information Technology Co., Ltd., Beijing, China (http://www.novogene.com). The clustering of the index-coded samples was performed on a cBot Cluster Generation System using TruSeq PE Cluster Kit v3-cBot-HS (Illumia) according to the manufacturer’s instructions. After cluster generation, the library preparations were sequenced on an Illumina Hiseq 4000 platform and 150 paired-end reads were generated.

### Data analysis

Raw sequence data reads in FASTA format were first processed using in-house Perl scripts. In this step, clean reads were obtained by removing adapters, polyN, and low-quality sequences from raw read data. At the same time, Q20 and GC content of the clean data were calculated. All downstream analyses were based on clean data with high quality. A Perl pipeline as described by Haas *et al*.^79^ was used for analyzing sequence data^79^. As suggested by Haas *et al*.^79^, if multiple sequencing runs are conducted for a single experiment, these reads may be concatenated into two files in the case of paired-end sequencing. The left files (read1 files) from all libraries/samples were pooled into one large left.fq file and the right files (read2 files) into one large right.fq file. Transcriptome assembly was accomplished based on left.fq and right.fq using Trinity^47^, with min_kmer_cov set to 2 by default and all other parameters set as the default. We used TGICL^80^ followed by the phrap^81^ assembler to remove redundant Trinity-generated contigs.

### Functional annotation of the assembled unigenes

For annotation, unigenes were searched against the NCBI non-redundant (NR) protein database using the BLASTALL package (release 2.2.22) with an E-value threshold of 10^−5^ (E-value ≤ 10^−5^). Consensus unigene sequences were further aligned to protein databases such as KEGG^82^ and KOG^83^ using the BLASTX algorithm to retrieve proteins with the highest sequence similarity to the given sequences along with the putative functional annotations for the proteins. If the results from different databases conflicted, a priority order of Swiss-Prot was followed. For domain/family annotation, the predicted amino acid sequences were searched against the Pfam database^84^ using HMMER 3.0 with the ‘Best Match Cascade’ protocol. Gene Ontology (GO)^85^ terms for each unigene were assigned based on the best BLASTx hit from the Pfam and NR databases using Blast2GO software^86^, with a significance threshold of E-value ≤ 10^−5^.

### Differential gene expression analysis

Differential gene expression (DGE) analysis of *M. albus* was performed using the DESeqR package (1.10.1), which provides statistical routines for determining differential expression in digital gene expression data using a model based on a negative binomial distribution. The calculation of unigene expression used the FPKM method (Fragments Per kb per Million reads)^87^. FPKM was directly used to compare the difference of gene expression levels between genotypes. The resulting *P* values were adjusted using the Benjamini-Hochberg approach for controlling the false discovery rate^88^. Differentially expressed genes (DEGs) between N48 vs N46 (CuCubb vs cucubb), N49 vs N47 (CuCuBB vs cucuBB), N47 vs N46 (cucuBB vs cucubb) and N49 vs N48 (CuCuBB vs CuCubb) were then assessed by KEGG pathway analysis. We used KOBAS^89^ software to test the statistical enrichment of differential expression genes in KEGG pathways. We used K-means clustering to assign the mean expression value of each gene to the cluster whose center is nearest. Lloyd’s algorithm was used for these experiments^90^. Euclidean distance was used as distance metric; four partitions were used to generate the clusters. For each gene, the mean gene expression value over all input samples was subtracted.

### PPI (Protein Protein Interaction)

The sequences of the DEGs were blast (blastx) to the genome of a related species (the protein protein interaction of which exists in the STRING database: http://string-db.org) to get the predicted PPI of these DEGs. Then the PPI of DEGs in comparison of N48 vs N46 was visualized in Cytoscape^91^.

### Quantitative real-time PCR (qRT-PCR) and expression analysis

Aliquots of the total RNA extracted for RNA sequencing were used for quantitative real-time PCR experiments. Total RNA (1 µg) was used for first-strand cDNA synthesis with M-MuLV First Strand cDNA Synthesis Kit (Sangon Biotech Co., Ltd, Shanghai, China) according to the manufacturer’s instructions. Quantitative real-time PCR was performed using an ABI 7500 Real-Time PCR System with 5 µl SYBR Green PCR Master Mix (Applied Biosystems, UK) following the manufacturer’s instructions. Gene-specific primers, shown in Table S11, were designed using Primer Premier 6.0 software. The *M. albus* β-tubulin gene was selected as the internal control gene. The thermal cycling conditions were as follows: 95 ºC for 5 min, followed by 40 cycles of 95 ºC for 5 s and annealing and extension at 60 ºC for 1 min. Melting curve analysis with 95 °C for 15 s, 60 °C for 1 min, and 95 °C for 15 s was performed to produce a dissociation curve for verification of the amplification specificity^92^. The relative expression levels of the selected unigenes were normalized to β-tubulin and calculated using the 2^−∆∆^Ct method. Each sample, including three replications with two technical replications, was used for real-time PCR analysis.

### Statistical analysis

All of the experiments used for data comparisons were repeated three times. The statistical analysis was performed using variance (ANOVA) followed by Duncan’s new multiple range test with SPSS version 20.0. The significance level is *P* < 0.05.

## Electronic supplementary material

Supplementary Information

Supplementary Table S2

Supplementary Table S3

Supplementary Table S4

Supplementary Table S5

Supplementary Table S6

Supplementary Table S7

Supplementary Table S8

Supplementary Table S9

Supplementary Table S10

Supplementary Table S11
