# Supplementary material for: Transcriptomic profiling of Melilotus albus near-isogenic lines contrasting for coumarin content
Source: Sci Rep. 2017 Jul 4;7:4577. doi: 10.1038/s41598-017-04111-y (PMC5496894; doi:10.1038/s41598-017-04111-y)
Supplement: Supplementary file 1 — Supplementary Information [file 41598_2017_4111_MOESM1_ESM.pdf]

Supplementary Information

Transcriptomic profiling of *Melilotus albus* near-isogenic lines contrasting for coumarin content

Kai Luo, Fan Wu, Daiyu Zhang, Rui Dong, Zhichao Fan, Rui Zhang, Zhuanzhuan Yan, Yanrong Wang*, Jiyu Zhang*

State Key Laboratory of Grassland Agro-ecosystems, College of Pastoral Agriculture Science and Technology, Lanzhou University, Lanzhou 730020, China

*Corresponding author: Yanrong Wang and Jiyu Zhang

E-mail: yrwang@lzu.edu.cn; zhangjy@lzu.edu.cn

List of Supplementary Information

Figure S1 Histogram of sequence length distribution of the assembled unigenes and transcripts in *M. albus.* . The bar chart shows length distribution of the unigenes (blue) and transcripts (red). The x-axis indicates a unigene length interval from < 300 bp to > 2000 bp. The y-axis indicates the number of unigenes of each given sequence length.

Figure S2 Venn diagram of the number of unigenes annotated by BLASTx (E-value ≤ 10^{-5}) against protein databases. The number in the circles indicates the number of unigenes annotated by single or multiple databases.

Figure S3 Results summary for sequence-homology search against NCBI NR database. (A) Similarity distribution of the closest BLASTX matches for each sequence. **(B)** A species-based distribution of BLASTX matches for sequences. **(C)** E-value distribution.

Figure S4 Functional classifications of the assembled unigenes. (A) Histogram of gene ontology (GO) classification. The results are summarized under three main GO categories: Biological process, cellular component and molecular function. The x-axis indicates the subcategories, and the y-axis indicates the numbers of unigene; the unigene that areassigned the same GO subcategories presented as the same colour. **(B)** Histogram of euKaryotic Ortholog Group (KOG) classification. The unigenes were aligned to the KOG database to predict and classify possible functions. A total of 38,709 sequences were annotated and separated into 26 clusters

Figure S5 Kyoto Encyclopedia of Genes and Genomes (KEGG) classification of non-redundant unigenes of *M. albus.* (A) Cellular Processes; (B) Environmental Information Processing; (C) Genetic Information Processing; (D) Metabolism; (E) Organismal Systems. The numbers on the top of bar chart indicate the number of unigenes that were mapped in each given KEGG pathways.

Figure S6 Classfication based on categories of secondary metabolite biosynthesis. The numbers on the top of bar chart indicate the number of unigenes that were mapped in each given secondary metabolite biosynthesis pathway.

Figure S7 Protein-protein interaction network analysis. The protein-protein interaction map includes differentially expression genes from the comparison of N48 vs N46 blast (blastx) to the genome of a related species sucessfully. The functional network was created by Cytoscape Software. Box nodes represent genes, and gray lines between two nodes represent interactions between genes. In the network, the coumarin biosynthesis related genes and their neighboring genes were presented as yellow colour.

Table S1 Summary of assembly quality for *M. albus* RNA-Seq.

Table S2 List of annotated unigenes of *M. albus* transcriptome as compared to public databases.

Table S3 NR annotation of common expressed unigenes in five *M. albus* genotypes.

Table S4 GO annotation of *M. albus* unigenes.

Table S5 KOG annotation of *M. albus*putative proteins.

Table S6 KEGG pathway annotation of *M. albus* unigenes.

Table S7 Differentially expressed genes that overlapping in the comparisons of N48 vs. N46 and N49 vs. N47.

Table S8 K-means cluster of DEGs in five *M. albus* genotypes.

Table S9 Unignes potentially related to phenylpropanoid biosynthesis pathway.

Table S10 Coexpression and PPI network of commonly regulated candidate genes and neighboring genes in the comparison of N48 vs N46.

Table S**11** List of primers used in qRT-PCR and expression patterns of genes validated by qRT-PCR.

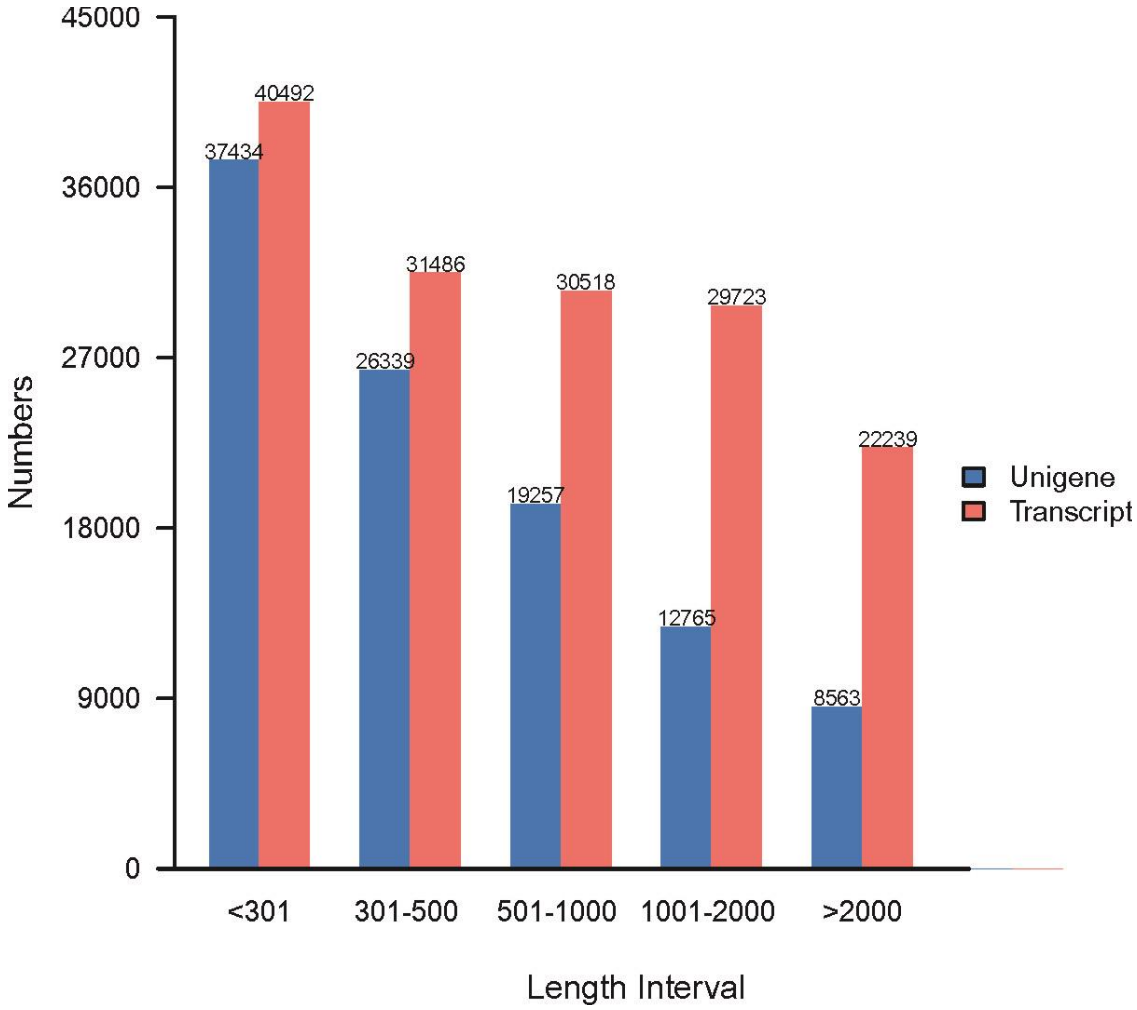

Figure S1 Histogram of sequence length distribution of the assembled unigenes and transcripts in *M. albus.* . The bar chart shows length distribution of the unigenes (blue) and transcripts (red). The x-axis indicates a unigene length interval from < 300 bp to > 2000 bp. The y-axis indicates the number of unigenes of each given sequence length.

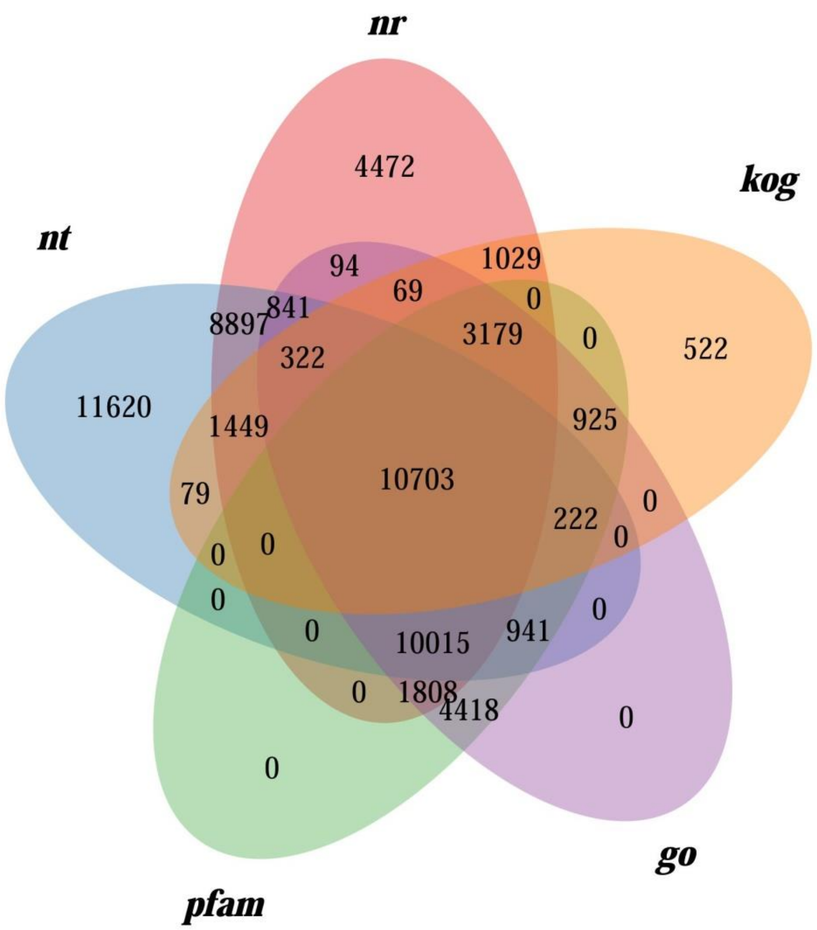

Figure S2 Venn diagram of the number of unigenes annotated by BLASTx (E-value ≤ 10^{-5}) against protein databases. The number in the circles indicates the number of unigenes annotated by single or multiple databases.

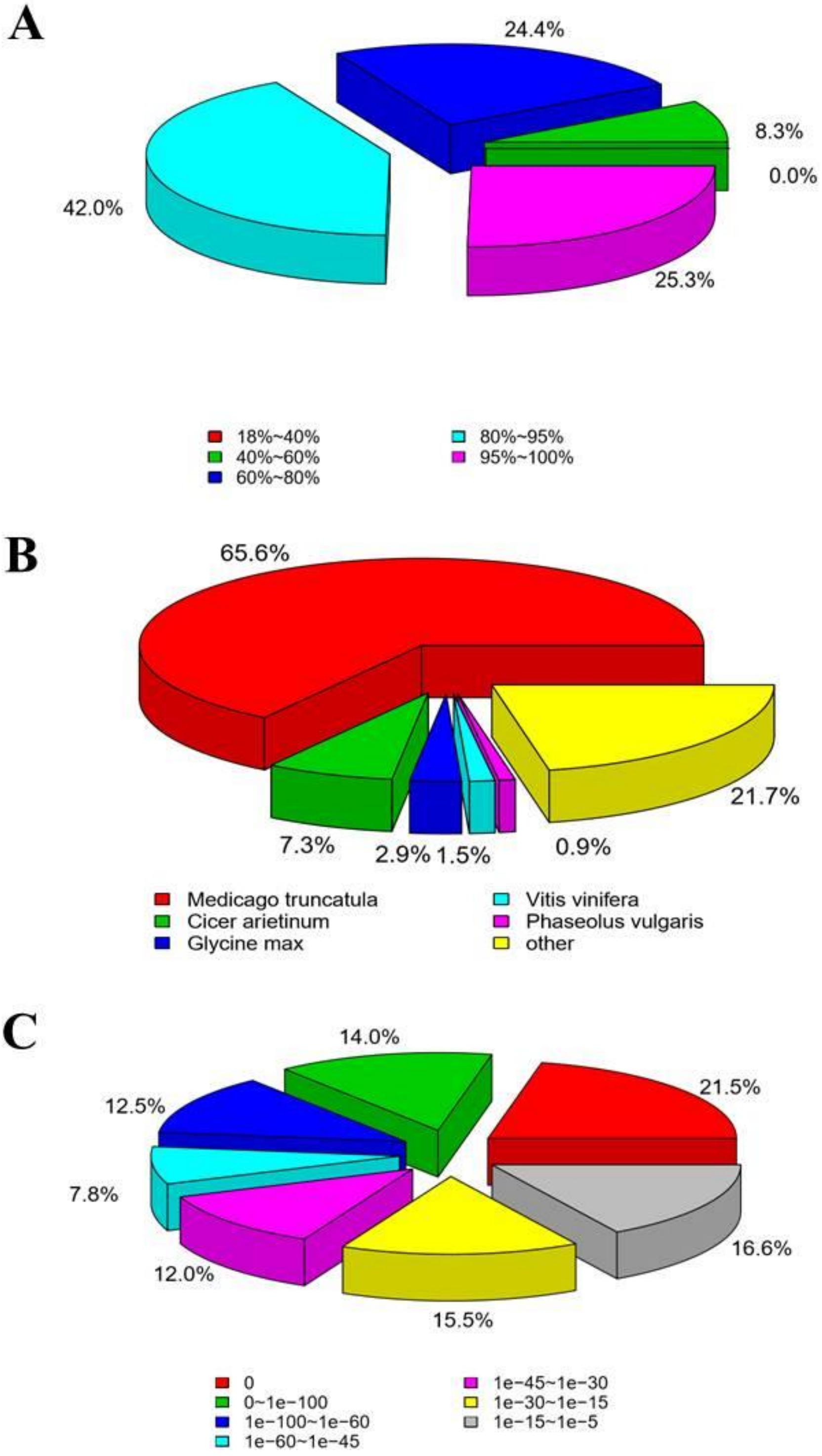

Figure S3 Results summary for sequence-homology search against NCBI NR database. (A) Similarity distribution of the closest BLASTX matches for each sequence. **(B)** A species-based distribution of BLASTX matches for sequences. **(C)** E-value distribution.

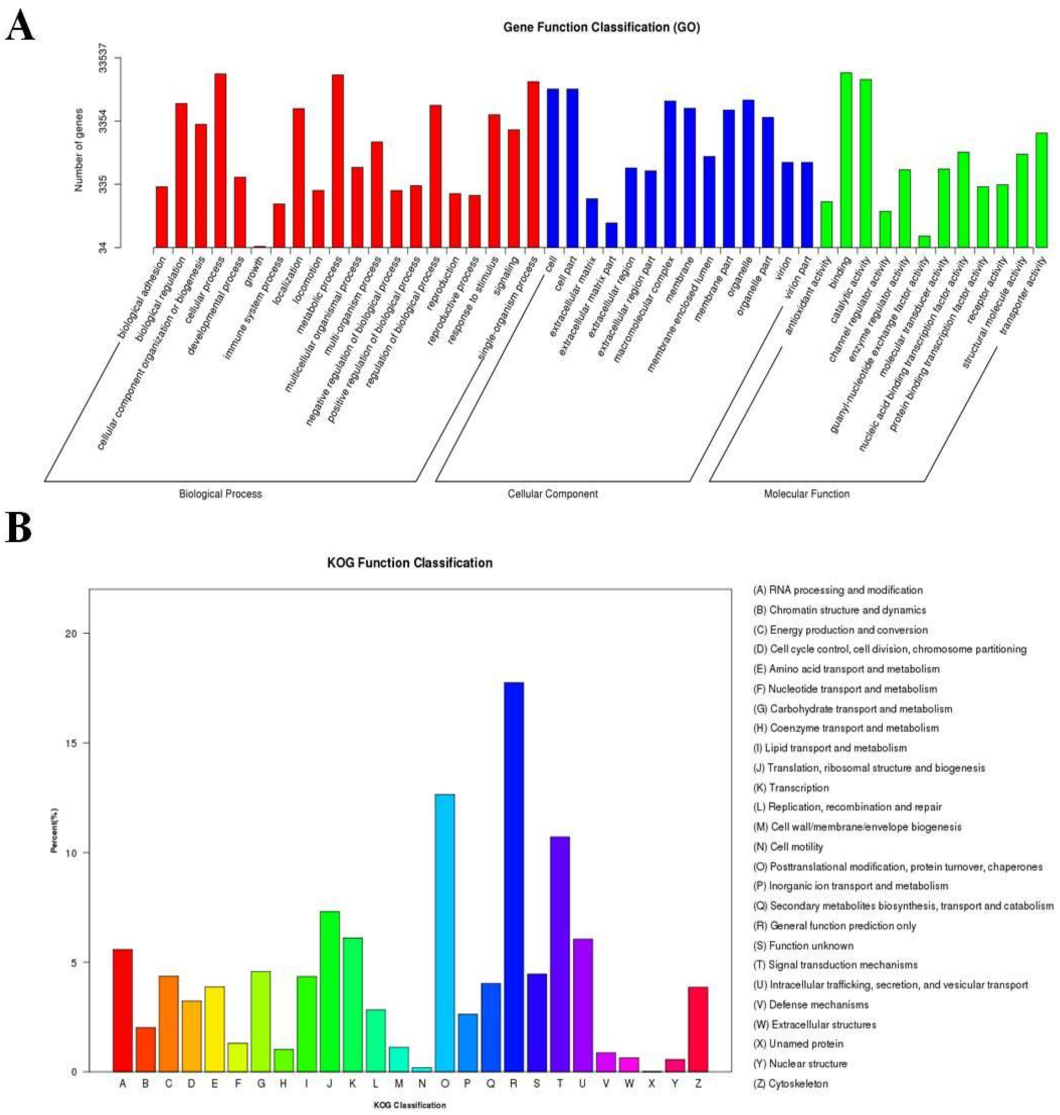

Figure S4 Functional classifications of the assembled unigenes. (A) Histogram of gene ontology (GO) classification. The results are summarized under three main GO categories: Biological process, cellular component and molecular function. The x-axis indicates the subcategories, and the y-axis indicates the numbers of unigene; the unigene that areassigned the same GO subcategories presented as the same colour. **(B)** Histogram of euKaryotic Ortholog Group (KOG) classification. The unigenes were aligned to the KOG database to predict and classify possible functions. A total of 38,709 sequences were annotated and separated into 26 clusters

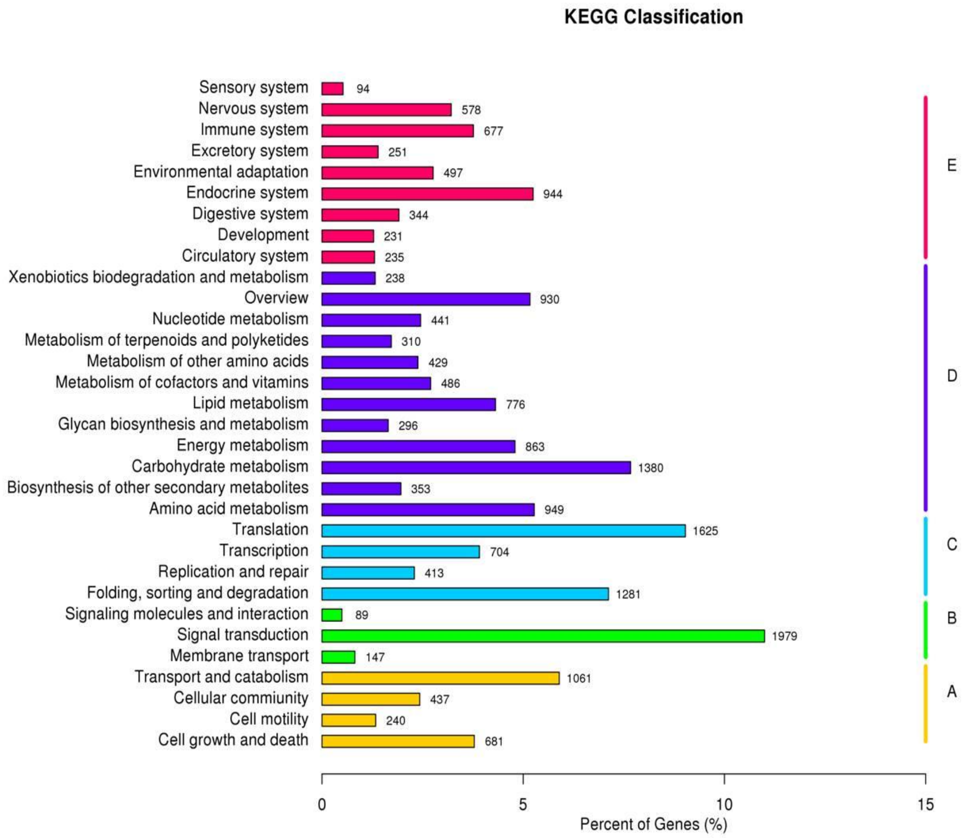

Figure S5 Kyoto Encyclopedia of Genes and Genomes (KEGG) classification of non-redundant unigenes of *M. albus.* (A) Cellular Processes; (B) Environmental Information Processing; (C) Genetic Information Processing; (D) Metabolism; (E) Organismal Systems. The numbers on the top of bar chart indicate the number of unigenes that were mapped in each given KEGG pathways.

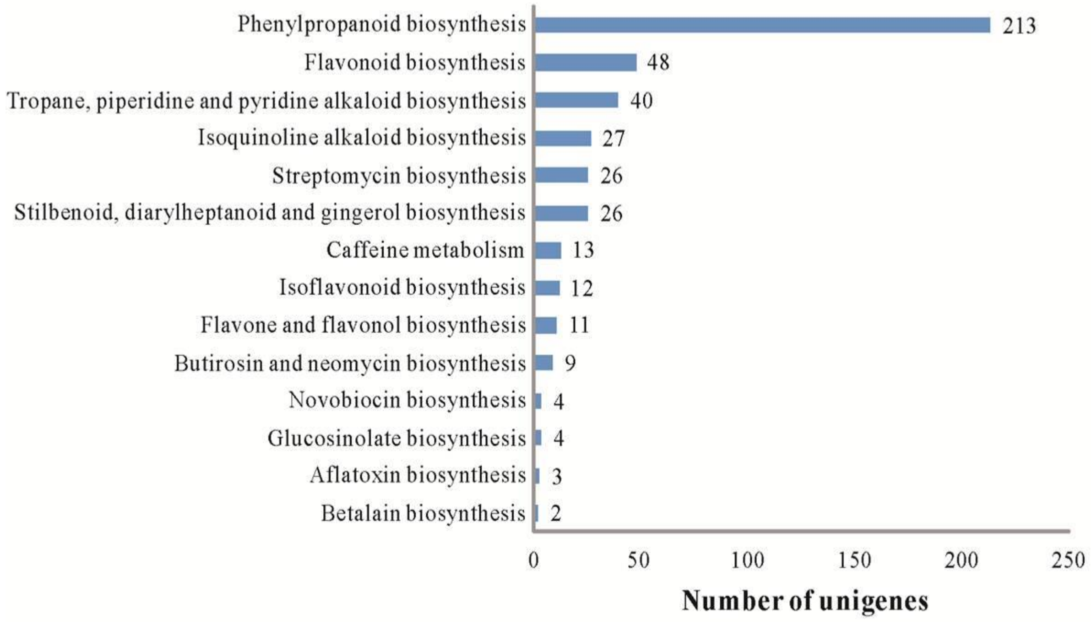

Figure S6 Classfication based on categories of secondary metabolite biosynthesis. The numbers on the top of bar chart indicate the number of unigenes that were mapped in each given secondary metabolite biosynthesis pathway.

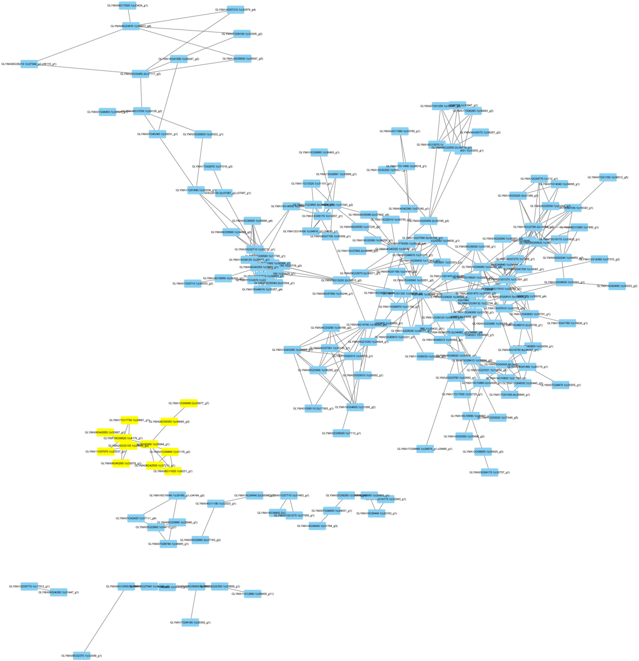

Figure S7 Protein-protein interaction network analysis. The protein-protein interaction map includes differentially expression genes from the comparison of N48 vs N46 blast (blastx) to the genome of a related species sucessfully. The functional network was created by Cytoscape Software. Box nodes represent genes, and gray lines between two nodes represent interactions between genes. In the network, the coumarin biosynthesis related genes and their neighboring genes were presented as yellow colour.

Table S1 Summary of assembly quality for *M. albus* RNA-Seq.

	**Transcripts**	**Unigenes**
**Mean Length (in bases)**	1,003	737
**Minimum Length (in bases)**	201	201
**Maximum Length (in bases)**	16,750	16,750
**N50 Length (in bases)**	1,782	1,297
**N90 Length (in bases)**	383	281
**200-500 bp number**	71,978	63,773
**500-1000 bp number**	30,518	19,257
**1000-2000 bp number**	29,723	12,765
**>2000 bp number**	22,239	8,563
**Total number**	154,458	104,358

Note：N50, N90 = length-weighted median contig length.
